# Grinding as Solvent-Free Green Chemistry Approach for Cyclodextrin Inclusion Complex Preparation in the Solid State

**DOI:** 10.3390/pharmaceutics10040189

**Published:** 2018-10-16

**Authors:** Mario Jug, Paola Angela Mura

**Affiliations:** 1 Department of Pharmaceutical Technology, Faculty of Pharmacy and Biochemistry, University of Zagreb, A. Kovačića 1, 10000 Zagreb, Croatia; mjug@pharma.hr; 2 Department of Chemistry ‘Ugo Schiff’, School of Human Health Sciences, University of Florence, Via Schiff 6, Sesto Fiorentino, 50019 Florence, Italy

**Keywords:** grinding, mechanochemical activation, cyclodextrins, inclusion complexes, amorphization, solid state interaction, solubility, dissolution

## Abstract

Among the different techniques proposed for preparing cyclodextrin inclusion complex in the solid state, mechanochemical activation by grinding appears as a fast, highly efficient, convenient, versatile, sustainable, and eco-friendly solvent-free method. This review is intended to give a systematic overview of the currently available data in this field, highlighting both the advantages as well as the shortcomings of such an approach. The possible mechanisms involved in the inclusion complex formation in the solid state, by grinding, have been illustrated. For each type of applied milling device, the respective process variables have been examined and discussed, together with the characteristics of the obtained products, also in relation with the physicochemical characteristics of both the drug and cyclodextrin subjected to grinding. The critical process parameters were evidenced in order to provide a useful guide for a rational selection of the most suitable conditions for an efficient inclusion complex preparation by grinding, with the final purpose of promoting a wider use of this effective solvent-free cyclodextrin inclusion complex preparation method in the solid state.

## 1. Introduction

Cyclodextrins (CDs) are well recognized multifunctional excipients able to increase solubility, dissolution rate, chemical stability, and bioavailability of different drugs through the inclusion complex formation [1,2]. Furthermore, CD encapsulation could reduce or prevent irritation and other side-effects of the drugs, enhance drug permeation across the biological membranes, prevent drug-to-drug and drug-to-excipient interactions, favorably modify organoleptic characteristics of included molecule and even convert liquid and volatile compounds in technologically more acceptable free-flowing powders [2,3,4,5,6,7]. Finally, CDs are often introduced into a wide range of advanced drug delivery formulations, ranging from monolithic to micro and nano-particulate ones, in order to target and control the drug release kinetic in accordance with the therapeutic needs [8,9,10,11]. In some cases, addition of a third component, such as polymers, organic acids, metal ions, or lipids could additionally enhance CD efficiency through a ternary complex formation [1]. Currently, CD technology has been applied in the development of at least 49 pharmaceutical products available worldwide [12]. Numerous kinds of CDs are commercially available, including naturally occurring α-cyclodextrin (αCD), β-cyclodextrin (βCD) and γ-cyclodextrin (γCD) and their numerous chemically-modified derivatives [2]. The hydrophilic CD derivatives with pharmaceutical relevance comprised in this review include hydroxypropyl-α-cyclodextrin (HPαCD), hydroxypropyl-β-cyclodextrin (HPβCD), hydroxyethyl-β-cyclodextrin (HEβCD) crystalline dimethyl-β-cyclodextrin (DIMEB), randomly methylated-β-cyclodextrin (RAMEB), sulphobuthylether-β-cyclodextrin sodium salt (SBEβCD), trimethylammonium-β-cyclodextrin (TMAβCD) and hydroxypropyl-γ-cyclodextrin (HPγCD) as well as CD polymers like β-cyclodextrin-epichlorohydrin insoluble polymer (βCD-EPI), β-cyclodextrin-epichlorohydrin soluble polymer (βCDEPS), and carboxymethyl-β-cyclodextrin-epichlorohydrin soluble polymer (CMβCD-EPS). Hydrophobic CD derivatives, such as triacetyl-β-cyclodextrin (TAβCD) and triacetyl-γ-cyclodextrin (TAγCD), are also included.

In recent years, numerous review papers were published, providing extensive data about the CD chemistry, toxicological and regulatory issues, mechanisms of the inclusion complex formation and changes of the included molecule solubility, chemical stability, permeability, bioavailability, and pharmacokinetics [1,2,3,4,6,7,8,9,11,13,14]. The analytical techniques used for CD complex characterization in solution and in the solid state have been also reviewed [15,16,17,18]. However, to the best of our knowledge, a comprehensive review of the available techniques for preparation of the inclusion complexes in the solid state is missing. The selection of the preparation method of the inclusion complex in the solid state is one of the critical steps in the development of a CD-based product, as it could determine the overall properties and functionality of the developed formulation [19,20]. It has been shown that an efficient CD complexation of the drug provides significantly faster dissolution rate and greater bioavailability enhancement than the corresponding samples, where only partial or no complexation (such as in the simple physical mixture) is achieved [21].

The main techniques available for the CD inclusion complexes preparation in the solid state can be grouped as follows:

1. *Methods in solution*, where drug and CD are dissolved in water or organic solvent/water mixtures, with pH and temperature adjusted to achieve maximum interaction between the components. In the case of B_s_-type complexes, mainly occurring with natural CDs, the product is isolated by crystallization [14], whereas in the case of A-type complexes, the solvent is removed by an adequate drying technique, such as coevaporation under reduced pressure (COE) [22,23], spray-drying (SPD) [24,25], or freeze-drying (FD) [26,27]. The drawbacks of such methods are in high consumption of time, energy and organic solvents (such as ethanol and methanol) whose complete removal from the final solid product could be quite costly and challenging, due to the ability of such solvents for the inclusion complex formation [28], that could lead to toxic effects. The use of supercritical fluid technology (SPF) could be also classified as a method of complex preparation in solution [29,30]. While, in this case, the use of organic solvents is in general avoided, this method requires the availability of specific and costly equipment.
2. *Methods in semisolid state*, where the drug-CD physical mixture is kneaded with a small volume of water or ethanol/water mixture to obtain a homogeneous pasty product and continued until a back a powder is gotten and then dried to completely remove the solvent (KN) [31,32]. However, such a method often results in only partial drug/CD complexation [14].
3. *Methods in the solid state*, where CD complexation is achieved by microwave irradiation (MWI) [33] or by gentle heating at temperatures below the fusion point of the compounds in a sealed container, eventually in the presence of a minimum water amount, according to the so-called “sealed-heating” method (SH) [34,35] or by mechanochemical activation through grinding (GR) with different types of mills [36,37] of the drug/cyclodextrin physical mixture. The drawback of both MW and SH technologies is in the possibility of drug degradation during microwave irradiation or heating [38]. Grinding, on the other hand, offers the advantages to be a simple, fast and highly effective method for the preparation of drug/CD inclusion complexes in the solid state, generally not requiring the use of organic solvents [39]. This provides additional advantages, avoiding problems and limitations of solution-based techniques, such as solubility issues, solvent complexation, or solvolysis. Therefore, grinding represents an economically and environmentally desirable technology and it will be the focus of this review.

## 2. Mechanism of the Inclusion Complex Formation in the Solid State by Grinding

Mechanochemistry refers to reactions, normally occurring in the solid state, induced by the input of mechanical energy [40]. It represents a powerful tool in a variety of application fields, ranging from material engineering to nanoscience. Manual grinding, using mortar and pestle, or more efficient mechanical grinding, using ball mills, oscillating or vibratory mills, are the most common process able to induce mechanochemical transformations [39]. Besides being the tool for the basic particle size reduction, grinding has been transformed into a continuously expanding toolkit for synthesis and screening of different supramolecular and covalent materials, complementing traditional strategies based on solvent-based synthesis [41,42]. The popularity of grinding-based mechanochemical synthesis lies in its extraordinary success in constructing metal-ligand coordination bonds, as well as non-covalent interactions hydrogen bonds, halogen bonds, π···π arene stacking, etc., thus providing the mean not only to activate otherwise inactive reactants, but also to incorporate and systematically study supramolecular structure-templating effects in a solvent-free synthesis. This lead to application of grinding in the development of pharmaceutically important polymorphs, cocrystals, porous meta-organic frameworks, polymeric dispersions, and also inclusion complexes [39,42,43].

**Figure 1:**
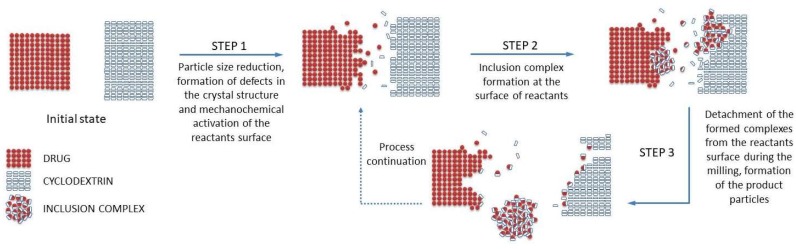
Schematic representation of the inclusion complex formation process in the solid state by grinding.

While there are no systematic studies investigating the exact mechanism of the inclusion complex formation occurring during grinding of a drug-cyclodextrin mixture, we are proposing a possible scenario, illustrated in Figure 1, based on the general three-steps mechanism described for mechanochemical reactions [44], and taking into account other processes occurring during the mechanochemical drug activation by grinding [39].

A drug/CD mixture when subjected to grinding receives mechanical energy pulse every time such material is being trapped between the colliding grinding media or between the mill wall and the grinding medium. If such an impact is of sufficient intensity, it results in a quasi-adiabatic local energy accumulation, giving origin to a metastable structure formation [39]. From a macroscopic point of view, the main part of the supplied energy is released by conversion into heat, which could facilitate the solid-state interactions between drug and CD. The thermally induced drug/CD interaction is a well-known phenomenon, often observed during the DSC analysis of the drug-CD systems [24,45]. Furthermore, the concentration of the strain field in particular crystal zones causes the crystal breaking, resulting in particle size reduction up to some critical threshold, as a result of the equilibrium between aggregation and comminution processes. This increases the overall surface available for the drug/CD interaction in the solid state. Further, energy supply leads to amorphization of the crystalline materials present in the treated mixture, usually starting on a thin surface layer and then propagating into the bulk, giving origin to an activated material formation [39]. It could be reasonably presumed that the inclusion complex formation takes place by reaction of activated materials at the surface of both drug and CD particles; this process could involve several intermediate phases, like a solid dispersion formation, which can gradually be transferred into a real inclusion complex in the solid state by molecular diffusion. As the grinding continues, the formed inclusion complex could be detached from the drug/cyclodextrin particles, forming separate particles, thus liberating the surfaces of drug/CD particles for the reaction continuation. Finally, grinding also provides an intense mixing and homogenization of the reactants, which further contribute to the drug/CD interaction in the solid state.

The above described technique is known in the literature as neat grinding [42,46]. In some cases, the addition of small sub-stoichiometric (catalytic) amounts of solvent induces more efficiently the interaction between the reactants during the grinding procedure, resulting in a higher yield of the product with the desired properties. Such technique, known as liquid-assisted grinding [42,46], was originally used in mechanochemical co-crystallization. In the field of cyclodextrin complexes, the liquid-assisted grinding is seldom used and may be an approach to prepare nanoparticles of poorly soluble drugs [47,48,49,50]. In other cases [51,52,53], the amount of added solvent was substantial, requiring an additional drying step of the paste-like product, thus corresponding to the kneading method, which is outside the scope of this review.

Grinding of a drug/CD mixture often produces amorphous products [31,32,33,34,47,51,54,55,56,57,58,59,60,61,62,63,64,65] or products containing only traces of crystalline drug [33,37,53,66,67,68,69,70,71,72,73,74,75,76], depending on the time and intensity of grinding and on the physicochemical properties of both drug and CD subjected to grinding. Several authors have demonstrated, by the use of ^13^C MAS CP/TOSS NMR spectroscopy, the actual inclusion complex formation in amorphous drug/CD products obtained by co-grinding; examples from the literature included two thiadiazole-based anti-Alzheimer drug candidates co-ground with βCD and HPβCD [27,37], a fentanyl/βCD [59], and a bisacodyl/βCD [54] co-ground system. However, even if the product contains a significant fraction of residual crystalline drug, it could be readily converted into inclusion complex upon dissolution in water, as demonstrated by Jablan et al. [77]. While the co-ground products of zaleplon with β-CD and polymeric β-CD derivative contained 51.10 and 12.05% of residual drug crystallinity (RDC), they showed significant improvement of the drug dissolution rate of 58.5 and 98.0% with respect to that of pure drug. This was attributed to the actual inclusion complexes formation upon dissolution, confirmed by ^1^H-NMR spectroscopy [77]. Grinding has often proved to be more effective than other techniques, such as kneading or coevaporation, in inducing complete amorphization of different drugs in combination with different CDs, such as clonazepam with βCD, HPβCD, and RAMEB [32], or oxaprozin in systems with both natural and derivative CDs [34], or naproxen with acetylated βCD and γCD [64]. Moreover, when compared to the complexes prepared by other techniques, amorphous products obtained by grinding in some cases presented superior solubility and dissolution properties, like that observed in case of co-ground products of oxaprozin with RAMEB, bile acids and chitosan [23] or with βCD or DIMEB [34], or of clonazepam with natural and derivative βCDs [32], or even of naproxen with acetylated CDs [64]. However, in other cases, complexes prepared by solvent based methods like coevaporation or freeze-drying showed superior dissolution properties and bioavailability [27,33,37,61,62,64,68,75]. Here, it must be taken into account that freeze-dried products, which often present superior performance over co-ground and coevaporated ones, have a highly porous structure that additionally contributes to the improvement in its dissolution rate and bioavailability [50,78].

## 3. Challenges in the Analytical Characterization of Cyclodextrin Complexes in the Solid State

Solid drug−CD inclusion complexes can be relatively complex systems, containing multiple amorphous and crystalline phases. In general, a combination of free and bound drug, as well as free and bound CDs may be present in a sample, especially in cases where the cyclodextrin type, the drug to CD ratio and the preparation conditions are not carefully optimized. Furthermore, the methods able to directly demonstrate an actual inclusion complex formation in the solid state are limited [16].

Differential scanning calorimetry (DSC) supported by X-ray powder diffraction (XRPD) are the analytical techniques most commonly used to characterize host-guest interactions in the co-ground products, providing essential data for selection of the optimal CD type and processing conditions for a given drug [16,24,32,66,79,80]. In some cases, conclusions made on the basis of DSC and XRPD results are further supported by Fourier-transform infrared spectroscopy (FTIR) and Scanning electron microscopy (SEM) [34,45,54,57,69,72,73,81]. The general approach is to compare thermal, spectral and morphological properties of the putative complex prepared by grinding to that of the drug-CD physical mixture and that of the pure compounds [16]. In many cases, RDC assessed as the ratio between the melting enthalpy of the drug in the co-ground sample (normalized to the drug content) and that of the pure drug, is used as an indicator of the solid-state drug-CD interaction in the different samples [24,25,31,32,36,47,73]. However, the drug RDC determination by DSC is only semiquantitative, and its accuracy and limits of detections (usually around 5%) can be significantly impaired by thermally induced interactions during the analysis [82]. Furthermore, the complete amorphization of the sample observed by both DSC and XRPD analysis is directly related only to the conversion of crystalline drug (or CD) to amorphous that could be a consequence of the formation of a true inclusion complex, as well as of numerous other phenomena occurred during the sample preparation and analysis (like formation of solid dispersion, release of crystalline water, moisture uptake, thermally induced interactions during DSC analysis, etc.) Therefore, DSC and XRPD data are able only to infer the inclusion phenomena in the analyzed sample, but are not a direct evidence of actual inclusion complex formation. Furthermore, XRPD cannot readily detect the presence of amorphous nonincluded (i.e., free) drug, while DSC can only detect its presence thorough glass transition events that can be quite challenging to detect in such a complex sample [83].

Another possible approach that could gave some insight on the amounts of included and nonincluded drug in the co-ground product is based on the different solubility in the organic solvents of highly polar drug-cyclodextrin inclusion complex and the lipophilic drug [84]. This could allow the extraction and quantification of the non-included drug from the sample. However, this approach requires sufficient difference in the solubility of the compounds in a given organic solvent, which is often not easy to achieve, due to a fact that the drug tends to “escape” from the central cavity of the cyclodextrin owing its higher affinity for the less polar organic solvent. Therefore, this method is rarely employed in everyday research.

Structural information about drug-CD complexation could be obtained by 1D solid-state NMR (ssNMR), However, the exact interpretation of the subtle chemical shift changes observed in the spectrum of the putative complex requires an adequate reference material. On the other hand, 2D ssNMR experiments offer more detailed data about the complex structure by directly probing interaction between specific NMR-active nuclei within the drug and the CD molecule [83]. For example, 2D ^1^H-^13^C cross-polarization heteronuclear correlation experiment (CP-HETCOR), which detects dipolar interaction between ^1^H and ^13^C nuclei, has been successfully applied to demonstrate the actual inclusion complex formation in the amorphous 1:1 complex of βCD and novel *Helicobacter pylori* eradicating agent (TG44) prepared by grinding [85]. The approach seeks to employ more sensitive nuclei such as ^1^H, ^19^F, and ^31^P whenever possible (depending on the drug structure) and uses ^13^C observation only when needed given the low drug loading typical of solid drug−CD complexes. Furthermore, the potential of ^19^F DP-MAS spectroscopy to quantitatively analyze the levels of included and free drug in a co-ground difunisal-βCD complex at 1:2 molar ratio was shown [83]. However, the method complexity, together with the requirement of particular expertise and costly equipment restrict the everyday use of such methodology.

## 4. Process Variables of the Inclusion Complex Formation in the Solid State by Grinding

While preparing drug/CD inclusion complexes in the solid state by grinding, it is important to understand and control the process variables. In general, the energy input, grinding time, grinding temperature, volume, as well as filling degree of the grinding jars are considered as the most important process variables. However, the influence of these variables is strictly related to the type of the milling device selected, which has a substantial effect on the efficiency of the grinding process and the quality parameters of the obtained product. For example, in the case of high energy vibrational mills, milling time and frequency are perhaps the easiest variables to control by adjusting the mill setup, while the temperature at which the milling is performed is not easy to control, since the reaction vessels heat up due to collisions occurring during milling. Parameters affecting the grinding temperature include filling degree, volume of the grinding balls, sample and vessel size, as well as the applied oscillating frequency. Higher frequency increases the velocity of the balls, increasing their energy, while prolonged milling time leads to increased energy input. However, the energy transfer upon collision cannot be directly controlled and it depends on volume of the grinding balls, degree of grinding jars filling (i.e., batch size), material from which the milling accessories are made, etc. [39,42,86].

The aim of this review is to provide a systematic overview of the most commonly used grinding devices and conditions applied, in order to provide a useful guideline for a rational selection of the most suitable conditions for an efficient inclusion complex preparation by grinding. With this purpose, the characteristics of both the drug and the CD will also be considered and put in line with the characteristics of the products obtained.

### 4.1. Inclusion Complex Preparation by Mortar and Pestle Grinding

The mortar and pestle has been the principal instrument of grinding science since the Stone Age and it is still useful today in mechanochemical synthesis [86]. While the energy input is low and difficult to quantify, grinding using pestle and mortar was efficiently applied as a cyclodextrin complex preparation method in the solid state and some examples from the literature are shown in Table 1. As it could be seen from the presented data, manual grinding leads to only partial drug complexation, especially in the cases where crystalline natural CDs are employed as complexing agents [70,87,88]. When amorphous CD derivatives were used, amorphous products were obtained [57,58,74,88]. RAMEB appears to be especially efficient in drug amorphization during manual grinding, even in cases of drugs with relatively high melting temperature (indicating relatively firm crystal lattice), like trimethoprim, sulphadiazine, and sulphamethoxazole (Table 1), where complete amorphization and probable inclusion complex formation occurred after only 15 min of grinding [88]. Surprisingly, in the case of gemfibrozil, a drug with relatively low melting point (indicating less stable crystal lattice), a longer grinding time was needed to obtain complete drug complexation with RAMEB (Table 1), as demonstrated by FTIR analysis [57]. This opposite result could be related to the different sample batch sizes, as well as to different mortar and pestle dimensions used for preparation, which are not described in those papers.

Especially interesting is the example of naproxen complexation by manual co-grinding with soluble and insoluble βCD-epichlorohydrin polymers (βCDEPS and βCDEPI, respectively), whose performance was almost comparable to that of RAMEB, previously found as the best carrier for naproxen; between the two polymeric derivatives, βCD-EPI presented higher amorphization power during the manual co-grinding process and the 10/90 (*w*/*w*) drug–carrier co-ground product exhibited the best dissolution properties, giving a dissolution efficiency about 30 times higher than that of naproxen alone [74].

### 4.2. Inclusion Complex Preparation by Grinding in High Energy Vibrational Mills

**Figure 2:**
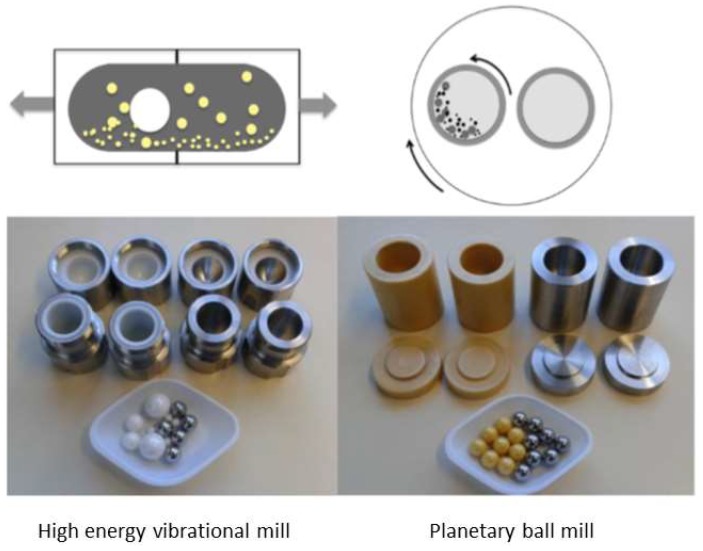
Most commonly used ball-milling equipment and milling media: High-energy vibrational mill: (**top**) Shaking direction, (**bottom**) ZrO_2_ and stainless-steel milling jars (10 mL) and balls. Planetary ball mill: (**top**) Movement description, (**bottom**) ZrO_2_ and stainless-steel milling vessels (12 mL) and balls. Adapted with permission from Reference [41]. Copyright (2017) American Chemical Society.

A typical high-energy vibrational mill, also known as mixer mill, is made of a toroidal shaped bowl or metal discs carrying separate cylindrical grinding jars connected to an engine. When the engine runs, the grinding jars perform radial oscillations in a horizontal position (Figure 2), while the inertia of the grinding balls causes them to impact with high energy on the sample material at the rounded ends of the grinding jars. Additionally, particle matter collisions and ball sliding further contribute to the mechanical energy supply, leading to sample pulverization and consequent mechanochemical activation. Furthermore, the movement of the grinding jars combined with the movement of the balls result in an intensive mixing of the sample. The mixing degree can be increased even further by using several smaller balls [39,89]. Other mill setups are also available, where a grinding vial is shaken in a complex motion that combines back-and-forth swings with short lateral movements in the shape of a ∞ sign [90]. The materials used for the construction of both vials and balls are hardened steel, stainless steel, tungsten carbide, agate, alumina ceramic, zirconia ceramic, silicon nitride, polystyrene, methacrylate, polycarbonate, and Teflon^®^. Vibrational mills are operating batch-wise and are suitable for laboratory preparation of products on the gram scale, while continuous vibrational mills are suitable for both pilot-plant and industrial applications [39,91].

Some examples of binary CD complexes prepared by high-energy vibrational mills are presented in Table 2. It is generally recognized that the effect obtained by grinding in vibrational mills mainly depends on the frequency of the vibrating motion, the grinding media material, shape (usually balls), and density as well as the grinding media-to-charge ratio [39]. Regarding the vibrational frequency, values ranging from 15 to 25 Hz are usually applied (Table 2). The effect of the vibrational frequency on the CD inclusion complex formation in the solid state is not straightforward, but also depends on the type and properties of the CD used. For example, in case of ketoprofen/RAMEB equimolar mixture, complete drug amorphization and probable inclusion complex formation was achieved after 60 min of grinding at 15 Hz, while the same result was achieved after only 30 min grinding at 25 Hz [92]. Furthermore, a more intense drug-carrier interaction was caused by partial dehydration of RAMEB, leading to complete drug amorphization after only 10 min grinding at both 15 and 25 Hz. On the contrary, in the case of equimolar mixture with βCD, the final product was partially crystalline, regardless applied vibrational frequency, indicating only partial drug complexation [92]. Ketoprofen complexation with RAMEB achieved by co-grinding increased its intrinsic dissolution rate constant approximately 100 times, in comparison with the approximate 10 times increase obtained from ground mixtures with βCD [92].

Regarding the batch size and grinding media/charge ratio, those values are only seldom stated in the literature [31,36,66,69,76,80,93]. It is obvious that the charging level of grinding jars is an important parameter that defines the free space available for the ball trajectories, thus defining their kinetic energy that could be transferred to the treated sample. It is generally accepted that the grinding jars should be filled up to 25% of their volume, taking into account both the volume of the sample and that of the grinding balls [91]. In general, the milling time necessary to achieve a complete product amorphization and probable inclusion complexation in the solid state varies between 30 and 60 min (Table 2), depending on the physicochemical characteristics of both drug and CD subjected to grinding as well as on the drug-to-CD ratio. However, in some cases, the successful amorphization was achieved in relatively shorter grinding times [31,93].

In general, co-grinding with crystalline CD derivatives leads to only partial drug complexation (i.e., formation of partially crystalline products) [32,77] or requires longer grinding time until complete product amorphization [31], which is generally accepted as an indication of inclusion complex formation in the solid state. On the other hand, hydrophilic βCD derivatives are more effective than natural βCD in establishing solid-state interactions with the drug by grinding. This could be clearly illustrated by the example of oxaprozin complexation with βCD, DIMEB, and RAMEB obtained by grinding, where amorphous RAMEB proved to be the most effective in performing solid-state interactions and in improving drug wettability and dissolution properties [34]. In case of co-ground complex with RAMEB, the percent of dissolved drug dose increased 7.2 times, while in the case of DIMEB and βCD, an increase of 4.4 and 1.9 times was observed, respectively. This example further evidences the superior performance of RAMEB for inclusion complex formation induced by grinding, which was also observed in the case of co-ground complexes of clonazepam [32] and ketoprofen [92]. On the other hand, in the case of co-ground complexes of loratadine [29] and praziquantel [36], amorphous HPβCD was a more efficient complexing agent than the parent βCD.

From the examples reported in Table 2, it appears evident that, among the different CD derivatives, polymeric CDs also proved to be very effective as co-grinding additives, producing amorphous complexes with enhanced dissolution properties [47,74,77,79,81]. Furthermore, co-grinding with βCD-EPI enhanced antibacterial activity of triclosan against *Streptococcus mutans*, that has a major role in etiology of caries [79]. A particularly interesting example of application of polymeric CDs in drug complexation by grinding was published by Cirri et al. [47]. Authors used βCD-EPI and CMβCD-EPI to increase the solubility of ketoprofen by inclusion complex formation through grinding in high vibrational mill, as a first phase in the development of a new delivery system consisting in ‘‘drug–in CD–in nanostructured lipid carriers’’ for topical delivery of this drug. Grinding was performed both in dry conditions and in the presence of 10% of water. The water presence during co-grinding markedly affected the drug/CD interaction and the grinding time necessary to obtain amorphous products. In the case of βCD-EPI, water reduced the grinding time needed for complete drug amorphization from 60 to 30 min (Table 2), while in the case of CMβCD-EPI an opposite effect was observed, with a grinding time of 120 min for obtaining an amorphous product, compared to only 30 min in dry conditions [47]. When such amorphous products were dissolved in water, drug nanoparticles were observed, with the size of 436 and 300 nm for βCD-EPI and CMβCD-EPI, ground in dry conditions. For the same products ground in the presence of 10% of water, mean particle size was 1275 and 1581 nm, respectively, clearly demonstrating different drug/CD interaction patterns during grinding in the presence and absence of water. Furthermore, this clearly highlights the possibility of drug/CD grinding as a mean of drug nanoparticles formation in case of poorly soluble compounds.

The mechanism of fine drug particles formation by co-grinding with CDs was studied in detail by Wongmekiat and coworkers [48,49,50], who demonstrated the critical role of the water content on yield and dimensions of formed drug nanoparticles, together with the grinding time, and the drug/CD ratio. In case of pranlukast hydrate/βCD systems, the highest yield of drug nanoparticles formation, with 50 nm in size, was obtained by 10 min grinding of 1:2 mol:mol drug/CD and 10% of water; a similar result was obtained with αCD and γCD, at the same drug-to-CD ratio and grinding time, but with a water level of 10 and 20%, respectively [49]. Based on the same concept, nanoparticles of indomethacin, furosemide, and naproxen were also prepared [50].

Grinding in high-energy vibrational mills was also applied to prepare complexes of drugs with hydrophobic CD derivatives. Some examples from the literature are given in Table 2. In case of metformin hydrochloride [72] and prilocaine hydrochloride [25], grinding with TAβCD resulted in only partial drug/carrier interaction. While crystalline TAβCD was converted into metastable amorphous form, both drugs partially retained their crystallinity in products co-ground for 30 min. On the contrary, spray drying from an ethanolic solution yielded in both cases amorphous products that substantially sustained the release of the complexed drug. In case of co-ground products of metformin hydrochloride and prilocaine hydrochloride, complete drug dose dissolution was observed after 120 and 30 min, respectively, while spray-dried products of both drugs presented a mean dissolution time of 420 min, clearly indicating a more intense solid-state interaction between the drug and the carrier obtained by the spray-drying technique.

Furthermore, grinding in high energy mills also appeared as a highly effective means to prepare ternary cyclodextrin/drug complexes where the ternary component could include hydrophilic polymers, phospholipids (egg phosphatidylcholine, EPC), bile acids or salts as well as small organic molecules, like amino-acids and hydroxy-acids. Some selected examples are presented in Table 3. The 100% increase in dissolution efficiency observed in the ternary GR product of naproxen with HPβCD and PVP has been attributed to an enhancement in the disorder of the system structure, i.e., to mechanochemical activation, optimized by the joint presence of the amorphous polymer and CD [78]. On the other hand, the improved drug dissolution properties of naproxen ternary GR systems with HPβCD and arginine with respect to the binary GR products has been explained with a synergistic effect between CD and the basic amino-acid [62]. The formation of a ternary complex has been proved in ternary GR systems of econazole with αCd and malic acid, responsible for the higher drug dissolution properties than the corresponding binary GR systems [75]. In the case of dehydroepiandrosterone (DHEA), the presence of glycine together with αCd at the 1:2:3 drug:CD:amino-acid molar ratios allowed the obtainment of a completely amorphous ternary GR system after 60 min grinding, while only partial amorphization was obtained in the corresponding binary GR systems [94]. It was found that the presence of glycine facilitated the structure destruction and loss of drug crystallinity during the co-grinding process, reducing the time and the milling vibration frequency necessary for achieving complete DHEA amorphization, probably by lowering the threshold for the system mechanochemical activation in virtue of its amphiphilic nature [88].

To the best of our knowledge, the only example of production-scale preparation of an inclusion complex by grinding was performed by Vectorpharma Spa of Trieste, Italy, that prepared βCD complexes of nimesulide, an antiinflammatory agent, by high energy milling. This is one of the limited examples of production-scale mechanochemistry. The complexation of nimesulide βCD was performed in a high-energy vibration mill on pilot (0.5–2 kg) and production (20–50 kg batch) scales with the optimum processing time of a modest 3.5 h. The RDC, taken as an indicator of batch-to-batch reproducibility was consistently 3–7% [96].

### 4.3. Inclusion Complex Preparation by Grinding in Planetary Mills

Planetary mills consist of a circular basement rotating around its main symmetry axis, carrying one or more grinding vials arranged eccentrically (Figure 2). The vials are co or counter-rotating with respect to the basement, subjecting the grinding balls in the grinding jars to superimposed rotational movements. The difference in speeds between the balls and grinding jars produces an interaction between frictional and impact forces, which releases high dynamic energies. The interplay between these forces produces a high and very effective degree of size reduction as well as the mechanochemical activation of the treated sample. Planetary mills are available both at laboratory or pilot-plant production scale and the grinding jars/balls could be made of different materials [39,97]. Some examples of drug/CD complexation obtained by grinding in planetary mills are presented in Table 4. In general, the mill is usually operated by alternating periods of milling with pause periods, in order to avoid sample overheating, with overall grinding time ranging from 1 to 5 h and rotation speed between 400 and 600 rpm. Such a setup resulted in the successful complex preparation in the solid state of different 1,2,4-thiadiazole anti-Alzheimer drug candidates [25,35]. However, it is here interesting to note that in both cases, although the inclusion complex formation by grinding was confirmed by ^13^C MAS CP/TSOO NMR, complexes prepared by freeze-drying presented higher dissolution rate and consequently better bioavailability [27,37]. Such a result can be attributed to the higher porosity of the freeze-dried sample compared to that obtained by grinding, which additionally contributed to the solubility of the complex formed; on the contrary, grinding lead to the formation of highly compacted particles [76,91]. Grinding in planetary mills operating at lower rotation rate and shorter time periods have also been applied, but resulted in only partial complexation of the treated drug [67], or required fine tuning of the drug-to-CD ratio [69] or even addition of a third compound to attain efficient CD complexation of the targeted molecule [98].

### 4.4. Inclusion Complex Preparation by Grinding in Desktop Tumbling Ball Mills

Desktop tumbling ball mills are rarely used to prepare CD inclusion complexes in the solid state. In this mill type, the sample to be treated and a large number of milling balls are loaded into a rotating cylinder (drum). Here the bulk energy transferred to the treated material mainly depends on the diameter of the drum and the applied speed [39]. In the case of desktop tumbling ball mills, which all have relatively small drum diameter, in order to attain mechanochemical activation of the sample and possible inclusion complexation in the solid state, longer milling times are required. Typical duration of the milling process is between 12 and 48 h, with a rotation speed in the range from 120 to 150 rpm [59,65,98]. For example, in the case of fentanyl, grinding the drug with βCD using 40 glass balls (20 with a diameter of 8 mm, and the other 20 of 12 mm), in 1:1 and 1:2 drug-to-CD molar ratios lead to amorphous products where the actual inclusion complexation was confirmed by solid state ^13^C-CP/MAS NMR spectroscopy [59]. Here, it is interesting to note that exposure of such product to 85% relative humidity resulted in drug recrystallization after one week, clearly underlying the need of a careful packaging of the obtained product, in order to preserve the properties obtained by co-grinding. In case of glibenclamide, co-grinding for 48 h at 1:2 drug to βCD molar ratio lead to amorphous complex formation, providing the complete drug dose dissolution in five min and chemically stabilizing the drug against elevated temperature and humidity mediated degradation [65]. Co-grinding of glimepiride for 48 h in desktop ball mill at 1:1 drug to βCD molar ratio resulted in amorphous inclusion complex formation as demonstrated by DSC and FTIR [96]. In this case, milling was performed either with alumina or glass balls. Interestingly, the complex prepared using glass balls showed a dramatically higher increase of drug solubility than that prepared by alumina-balls, indicating that the material of the milling balls may affect the mechano-chemical reaction between glimepiride and CD, due to physical and chemical factors, such as mobility, hardness, and surface characteristics of the milling balls. The authors suggested that alkaline components in the soda-lime glass balls have been probably incorporated into the ground mixture during the co-grinding process, contributing to an increase in the local pH, thereby increasing the overall drug solubility [98].

## 5. Conclusions

Grinding proved to be a versatile, easy to use, highly functional and eco-friendly approach for preparation of the CD inclusion complexes in the solid state. The operating conditions must be properly tuned taking into consideration the type of the milling device applied, as well as the physicochemical characteristics of both the drug and CDs subjected to grinding, in order to obtain a successful solid-state interaction between the components, thereby improving the unfavorable biopharmaceutical properties of the drug and enhancing its bioavailability and therapeutic efficacy. However, in spite of the wide use of this technique, the existing body of mechanistic knowledge about the drug-CD inclusion complex formation by grinding is still modest.

Therefore, this review was attempted to provide a systematic overview of the data currently available with the aim of becoming a starting point of further scientific investigations that will lead to a more profound understanding of the underlying mechanisms of this valid and useful technique, thus further favoring and expanding its successful industrial application.

## Figures and Tables

**Table 1:** An overview of drug/CD complexes prepared by manual grinding using mortar and pestle.

Drug	CD ^1^	Drug/CD Ratio ^2^	Grinding Conditions	Properties of the Obtained Product	Reference
Chloramphenicol (mp 155.23 °C)	β-CD	1:1	up to 120 min	Partial inclusion (ca. 32%) after 120 min grinding	[70]
Gemfibrozil (mp. 59.25 °C)	DIMEB	1:1	up to 35 min	Amorphous product after 35 min grinding	[57]
Naproxen (mp 153.4 °C)	βCDEPI βCDEPS	50/50, 20/80, 15/85, 10/90 (*w*/*w*)	up to 40 min	Amorphous product with enhanced dissolution properties	[74]
Naproxen (mp. 153.4 °C)	αCD maltohexaose	0.3–0.1 (*w*/*w*)	up to 30 min	Pseudo-inclusion complex formation with maltohexaose, partial interaction with αCD	[87]
Trimetoprim (mp 170 °C) Sulphadiazine (mp 260.6 °C) Sulphamethoxazole (mp 201 °C)	αCD βCD γCD RAMEB DIMEB	1:1	15 min	Amorphous products with RAMEB	[88]
Rifaldazine (mp 259 °C)	βCD	1:1	3 min trituration followed by 30 min grinding	Amorphous product, 4.4 times higher solubility; inclusion complexation confirmed by FTIR	[56]
Rifampicin (mp 259 °C)	HPβCD	1:1	3 min trituration followed by 30 min grinding	Amorphous product with 2.5 times higher solubility	[58]

^1^ the full name of CDs is given in the Introduction; ^2^ molar ratio if not otherwise stated; mp–melting point.

**Table 2:** An overview of binary drug/CD complexes prepared by grinding using high energy vibrational mills.

Drug	CD ^1^	Drug/CD Ratio ^2^	Grinding Conditions	Properties of the Obtained Product	Reference
Bupivacaine hydrochloride (mp 128.19 °C)	βCD βCD-EPI	1:1	30–60 min at 24 Hz, ambient conditions	Amorphous products with enhanced dissolution properties by GR with βCD-EPI; partially crystalline by GR with βCD	[81]
Clonazepam (mp 239.1 °C)	αCD HPαCD βCD HPβCD RAMEB γCD HPγCD	1:1	30 min at 24 Hz, ambient conditions	61.1 and 16.4% RDC for αCD and HPαCD GR, respectively; amorphous with other CDs; the most efficient was RAMEB (dissolution rate rank GR > COE ≈ KN >PM)	[32]
Daidzein (mp 336.4 °C) Genistein (mp 308.5 °C)	HPβCD SBEβCD	1:1	30 min at ambient conditions in 10 mL SS jars with two 7 mm SS balls	Partially crystalline products; SBEβCD more efficient as amorphizing agent	[66]
Econazole (mp 89.0 °C)	αCD	1:1	60 min at 24 Hz, ambient conditions, batch size 1 g	Partially amorphous system obtained by GR; completely amorphous complex by FD.	[75]
Econazole nitrate (mp 163.78 °C)	HEβCD HPβCD SBEβCD EPIβCD	1:1	15–60 min at 24 Hz, ambient conditions	RDC always decreased as a function of grinding time; amorphous product with HPβCD and SBEβCD after 60 min GR	[80]
Glyburide (mp 175.3 °C)	αCD βCD HPβCD RAMEB γCD	1:1	0–60 min at 24 Hz, ambient conditions	Amorphous products obtained in all cases after 60 min, but with different sensitivity to mechanochemical activation. No drug recrystallization during storage.	[73]
Indomethacin nicotinamide cocrystals (mp 128.5 °C)	βCD HPβCD	1:1	15 min	Partial complexation for GR products with βCD and HPβCD. Amorphous system with enhanced dissolution rate by COE with HPβCD	[68]
Ketoprofen (mp 94.6 °C)	βCD-EPI CMβCD-EPI	10:90 (*w*/*w*)	10–120 min at 24 Hz ambient temperature with or without 10% water	Complete amorphization by dry GR with βCD-EPI (60 min) and CMβCD-EPI (30 min). In the moist conditions complete amorphization after 30 min GR with βCD-EPI, 120 min with CMβCD-EPI.	[47]
Ketoprofen (mp 94.6 °C)	βCD RAMEB	1:1	10–60 min at frequency from 15 to 24 Hz and ambient temperature	Complete amorphization after 60 min GR at 15 Hz or 30 min at 25 Hz with RAMEB; partial crystalline systems with βCD regardless applied frequency.	[92]
Loratadine (mp 136.1 °C)	HPβCD	1:1 or 1:2	25 mL SS jar with two 15 mm SS balls, at 15 Hz up to 30 min. Batch size 0.2 g	Complete amorphization after 7 min GR with HPβCD at 1:1 ratio and after 15 min at 1:2 ratio. Inclusion complex formation verified by FTIR.	[93]
Loratadine (mp 136.1 °C)	βCD HPβCD	1:1	25 mL SS jar with two 15 mm SS balls at 15 Hz up to 30 min. Batch size 0.2 g	Complete amorphization after 7 and 15 min GR with HPβCD and βCD, respectively. Amorphization process followed zero-order kinetics.	[31]
Metformin hydrochloride (mp 231.0 °C)	TAβCD	1:1	30 min at 24 Hz and ambient conditions	Partially crystalline product by GR, completely amorphous by SPD and characterized with the most pronounced sustained release profile	[72]
Naproxen (mp 155.9 °C)	SBEβCD TMAβCD	1:1	30 min at 24 Hz and ambient conditions	Amorphous products by GR and FD; FD complex presented almost double dissolution efficiency. SBEβCD was the best carrier.	[61]
Naproxen (mp 155.9 °C)	TAβCD TAγCD	1:1	30 min at 24 Hz and ambient conditions	Amorphous products by GR and FD. FD complexes showed faster initial dissolution followed by decline due to supersaturation, not observed for GR products	[64]
Oxaprozin (mp 161.3 °C)	βCD DIMEB RAMEB	1:1	30 min at 24 Hz and ambient conditions	Partially crystalline product with βCD, amorphous with DIMEB and RAMEB. Drug dissolution rate increased 7.2, 4.4 and 1.9 times with RAMEB, DIMEB and βCD complexes, respectively.	[34]
Telmisartan (mp 265.3 °C)	βCD	1:2 or 1:3	65 mL SS jars with 3 SS ball (two of 6.4 and one of 12.8 mm) at 3.7 ball-to-powder ratio for 7, 15 and 30 min Grinding frequency not stated	Formation of new solid phases in all samples after 30 min GR; 19-fold increase of drug dissolution and rapid and effective antihypertensive effect in rat model	[69]
Triclosan (mp 59.48 °C)	βCD βCD-EPI	1:1	10–80 min at 24 Hz and ambient temperature	Complete amorphization after 60 and 80 min GR with βCD-EPI and βCD, respectively. Complexation with βCD-EPI enhanced drug dissolution and antimicrobial activity	[79]
Pranlukast hemihydrate (mp 192.1 °C)	βCD βCD 10.5 H_2_O	1:2, 1:1, or 2:1	10 min Grinding frequency not stated	βCD hydrated GR systems appeared as amorphous stiff mass; those with anhydrous βCD as fine crystalline powder. βCD hydrated GR systems dispersed in water formed a fine suspension (particle size 0.04–0.06 μm)	[48]
Pranlukast hemihydrate (mp 192.1 °C)	αCD βCD γCD	1:2	10 min with 0.75–20% of water Grinding frequency not stated	GR with βCD prepared with 13% of water almost completely transferred into fine drug particles after dispersion in water; similar behavior for αCD and γCD GR with 10 and 20% of water.	[49]
Praziquantel (mp 142.28 °C)	βCD HPβCD RAMEB SBEβCD	1:1	30 min at 25 HZ in 10 mL SS jars with two 7 mm SS balls; ambient conditions; batch size 200 mg	Partially crystalline product with βCD (RDC 61.63%), amorphous products with other CDs. GR with HPβCD showed 10 fold dissolution rate increase and acceptable chemical stability during storage; substantial drug degradation in other products.	[36]
Prilocaine hydrochloride (mp 169.9 °C)	TAβCD	1:1	30 min at 24 Hz and ambient temperature	Partially crystalline product by GR (RDC 28%), amorphous by SPD, with more pronounced sustained release.	[25]
Zaleplon (mp 185.27 °C)	βCD βCD-EPI	1:1	10–90 min at 24 Hz and ambient conditions	Superior performance of βCD-EPI vs. βCD (RDC 12.05 and 51.10%, respectively) and 25% faster dissolution rate. Formation of actual inclusion complexes after dissolution in water proved by ^1^ H-NMR	[77]

^1^ the full name of CDs is given in the Introduction; ^2^ molar ratio if not otherwise stated; mp–melting point; SS–stainless steel.

**Table 3:** An overview of ternary drug/CD complexes prepared by grinding using high-energy vibrational mills.

Drug	CD/Ternary Compound ^1^	Drug/CD/Ternary Compound Ratio ^2^	Grinding Conditions	Properties of the Obtained Product	Reference
Dehydroepiandrosterone (mp 150.9 °C)	αCD + glycine	1:1:2 or 1:2:3	60 min at 24 Hz, ambient conditions	Partially crystalline product at 1:1:2 and amorphous at 1:2:3 molar ratio. Superior performance of 1:2:3 complex confirmed in vivo	[94]
Econazol (mp 89.0 °C)	αCD + malic acid	1:1:1	60 min at 24 Hz ambient conditions, batch size 1 g	Partially crystalline ternary product prepared by GR; FD was amorphous with superior dissolution properties	[75]
Econazol nitrate (mp 123.12 °C)	SBEβCD + citric acid	1:1:1	60 min at 24 Hz and ambient conditions	Amorphous product with superior dissolution profile, without supersaturation phenomenon, as instead observed for binary GR complex	[80]
Ketoprofen (mp 96.5 °C)	βCD or RAMEB + EPC	20:76:4 (*w*/*w*)	15 or 30 min at 24 Hz and ambient conditions	Partially crystalline ternary complexes prepared by GR, amorphous when prepared by MWI that showed superior dissolution profile	[33]
Naproxen (155.9 °C)	HPβCD + L-arginine	1:1:1	60 min at 24 Hz and ambient conditions; batch size 500 mg	Enhanced dissolution of ternary complex prepared by GR, further dissolution increase of ternary complex prepared by COE	[62]
Naproxen (155.9 °C)	HPβCD + PVP	1:1 + 15% (*w/w*)	60 min at 24 Hz and ambient conditions	Amorphous binary and ternary GR systems; ternary showed 100% higher drug dissolution efficiency than the binary ones	[78]
Oxaprozin (mp 161.3 °C)	RAMEB + bile acids/salts + chitosan	1:1:1 + 0.0625% (*w*/*w*)	30 min at 24 Hz and ambient conditions	Amorphous ternary and quaternary products with enhanced dissolution and permeability. GR products showed superior performance than those prepared by COE and SH	[23]
Oxaprozin (mp 161.3 °C)	RAMEB + L-arginin	1:1:1	30 min at 24 Hz and ambient conditions	Amorphous products both in binary and ternary complexes; ternary with 10 times higher relative dissolution rate than binary complexes	[95]
Praziquantel (mp 142.28 °C)	HPβCD or RAMEB + malic acid	1:1:1	10 mL SS jars containing two 7 mm SS balls for 30 min at 25 HZ and ambient conditions; batch size 200 mg	Amorphous ternary complexes with solubility lower than corresponding binary ones. Ternary complex formation led to pronounced chemical degradation of the drug	[36]

^1^ The full name of CDs is given in the Introduction; ^2^ molar ratio if not otherwise stated; mp, melting point; SS, stainless steel.

**Table 4:** An overview of drug/CD complexes prepared by grinding using planetary mills.

Drug	CD ^1^	Drug/CD Ratio ^2^	Grinding Conditions	Properties of the Obtained Product	Reference
1,2,4-thiadiazole anti-Alzheimer drug candidate (mp not given)	βCD HPβCD	1:1	12 mL agate jar with 5 mm agate balls for 60 min at 600 rpm with pauses to prevent sample overheating	Inclusion complex formation confirmed by 13C MAS CP/TSOO NMR; FD complex presented higher bioavailability	[27]
1,2,4-thiadiazole anti-Alzheimer drug candidate (mp 50 and 102 °C)	βCD HPβCD	1:1	12 mL agate jar with 5 mm agate balls for 60 min at 600 rpm with pauses to prevent sample overheating	Inclusion complex formation confirmed by 13C MAS CP/TSOO NMR; FD complex showed higher solubility	[37]
Bisacodyl (mp 136 °C)	βCD	1:1	50 mL agate jar; agate balls (mixture of 15 × 10 mm, 55 × 5 mm, and 40 × 2 mm) for 5 h at 400 rpm alternating milling (5 min) and pause (1 min) periods to prevent sample overheating	Amorphous product with higher solubility than complexes prepared by FD and COE. Inclusion complexation confirmed by 13C CP/MAS NMR	[54]
Lipoic acid (mp 62.9 °C)	HPβCD + Na_2_CO_3_	1:1 and 1:1:1.2	25 mL jar and 12 mm balls (1/3 of the tank volume) for 120 min at 150 rpm	Partially crystalline binary system; amorphization achieved by addition of Na_2_CO_3_ that enhanced complexation by GR, increasing the compound solubility and chemical stability	[99]
Opipramol base (mp 100 °C)	βCD	1:1	12 mL jar for 10 min at 400 rpm	Partial amorphization with GR, complete by FD. Both products showed comparable increase of drug dissolution rate.	[67]
Telmisartan (mp 265.3 °C)	βCD	1:2 and 1:3	65 mL steel jar and 3 steel balls (two of 6.4 and one 12.8 mm) at 3.7 ball/powder ratio; milling time 7, 15 and 30 min	Amorphous systems at 1:2 and 1:3 ratio at 30 min GR	[69]
Zaltoprofen (mp 138 °C)	βCD cucurbit [7] uryl	1:1	50 mL agate jar with mixture of 10 × 10 mm–20 × 5 mm agate balls for 5 h at room temperature and 400 rpm, with alternate grinding (5 min) and pause (1 min) periods	Amorphous products in both cases with increased dissolution rate. GR of drug alone failed to improve its solubility	[100]

^1^ the name of CDs is given in the Introduction; ^2^ molar ratio if not otherwise stated ^1^; mp–melting point; SS–stainless steel.

## References

[1] Kurkov S.V., Loftsson T. (2013). Cyclodextrins. Int. J. Pharm..

[2] Jansook P., Ogawa N., Loftsson T. (2018). Cyclodextrins: Structure, physicochemical properties and pharmaceutical applications. Int. J. Pharm..

[3] Popielec A., Loftsson T. (2017). Effects of cyclodextrins on the chemical stability of drugs. Int. J. Pharm..

[4] Loftsson T., Brewster M.E. (2011). Pharmaceutical applications of cyclodextrins: Effects on drug permeation through biological membranes. J. Pharm. Pharmacol..

[5] Arima H., Higashi T., Motoyama K. (2012). Improvement of the bitter taste of drugs by complexation with cyclodextrins: Applications, evaluations and mechanisms. Ther. Deliv..

[6] Loftsson T., Brewster M.E. (2010). Pharmaceutical applications of cyclodextrins: Basic science and product development. J. Pharm. Pharmacol..

[7] Marques H.M.C. (2010). A review on cyclodextrin encapsulation of essential oils and volatiles. Flavour Fragr. J..

[8] Salústio P.J., Pontes P., Conduto C., Sanches I., Carvalho C., Arrais J., Marques H.M.C. (2011). Advanced Technologies for Oral Controlled Release: Cyclodextrins for Oral Controlled Release. AAPS PharmSciTech.

[9] Zhang J., Ma P.X. (2013). Cyclodextrin-based supramolecular systems for drug delivery: Recent progress and future perspective. Adv. Drug Deliv. Rev..

[10] Gref R., Duchêne D. (2012). Cyclodextrins as “smart” components of polymer nanoparticles. J. Drug Deliv. Sci. Technol..

[11] Adeoye O., Cabral-Marques H. (2017). Cyclodextrin nanosystems in oral drug delivery: A mini review. Int. J. Pharm..

[12] Wen H., Jung H., Li X. (2015). Drug Delivery Approaches in Addressing Clinical Pharmacology-Related Issues: Opportunities and Challenges. AAPS J..

[13] Duchêne D., Bochot A. (2016). Thirty years with cyclodextrins. Int. J. Pharm..

[14] Jones D.S., Dressman J.B., Loftsson T., Moya-Ortega M.D., Alvarez-Lorenzo C., Concheiro A. (2016). Pharmacokinetics of cyclodextrins and drugs after oral and parenteral administration of drug/cyclodextrin complexes. J. Pharm. Pharmacol..

[15] Mura P. (2014). Analytical techniques for characterization of cyclodextrin complexes in aqueous solution: A review. J. Pharm. Biomed. Anal..

[16] Mura P. (2015). Analytical techniques for characterization of cyclodextrin complexes in the solid state: A review. J. Pharm. Biomed. Anal..

[17] Ogawa N., Takahashi C., Yamamoto H. (2015). Physicochemical characterization of cyclodextrin-drug interactions in the solid state and the effect of water on these interactions. J. Pharm. Sci..

[18] Szente L., Szemán J., Sohajda T. (2016). Analytical characterization of cyclodextrins: History, official methods and recommended new techniques. J. Pharm. Biomed. Anal..

[19] Mura P., Faucci M., Manderioli A., Bramanti G. (1999). Influence of the preparation method on the physicochemical properties of binary systems of econazole with cyclodextrins. Int. J. Pharm..

[20] Mohit V., Harshal G., Neha D., Vilasrao K., Rajashree H. (2011). A comparative study of complexation methods for cefdinir-hydroxypropyl-β-cyclodextrin system. J. Incl. Phenom. Macrocycl. Chem..

[21] Miller L.A., Carrier R.L., Ahmed I. (2007). Practical considerations in development of solid dosage forms that contain cyclodextrin. J. Pharm. Sci..

[22] Desai N.S., Bramhane D.M., Nagarsenker M.S. (2011). Repaglinide-Cyclodextrin complexes: Preparation, Characterization and in vivo evaluation of antihyperglycemic activity. J. Incl. Phenom. Macrocycl. Chem..

[23] Maestrelli F., Cirri M., Mennini N., Zerrouk N., Mura P. (2011). Improvement of oxaprozin solubility and permeability by the combined use of cyclodextrin, chitosan, and bile components. Eur. J. Pharm. Biopharm..

[24] Jablan J., Szalontai G., Jug M. (2012). Comparative analysis of zaleplon complexation with cyclodextrins and hydrophilic polymers in solution and in solid state. J. Pharm. Biomed. Anal..

[25] Bragagni M., Maestrelli F., Mura P. (2010). Physical chemical characterization of binary systems of prilocaine hydrochloride with triacetyl-β-cyclodextrin. J. Incl. Phenom. Macrocycl. Chem..

[26] Jug M., Bečirević-Laćan M., Bengez S. (2009). Novel cyclodextrin-based film formulation intended for buccal delivery of atenolol Cyclodextrin-based film formulation for buccal delivery of atenolol. Drug Dev. Ind. Pharm..

[27] Promzeleva M., Volkova T., Proshin A., Siluykov O., Mazur A., Tolstoy P., Ivanov S., Kamilov F., Terekhova I. (2018). Improved Biopharmaceutical Properties of Oral Formulations of 1,2,4-Thiadiazole Derivative with Cyclodextrins: In Vitro and in Vivo Evaluation. ACS Biomater. Sci. Eng..

[28] Aree T., Chaichit N. (2003). A new crystal form of β-cyclodextrin–ethanol inclusion complex: Channel-type structure without long guest molecules. Carbohydr. Res..

[29] Huang Y., Zu Y., Zhao X., Wu M., Feng Z., Deng Y., Zu C., Wang L. (2016). Preparation of inclusion complex of apigenin-hydroxypropyl-β-cyclodextrin by using supercritical antisolvent process for dissolution and bioavailability enhancement. Int. J. Pharm..

[30] Rudrangi S.R.S., Kaialy W., Ghori M.U., Trivedi V., Snowden M.J., Alexander B.D. (2016). Solid-state flurbiprofen and methyl-β-cyclodextrin inclusion complexes prepared using a single-step, organic solvent-free supercritical fluid process. Eur. J. Pharm. Biopharm..

[31] Lin H.-L., Lin S.-Y., Lin C.-C., Hsu C.-H., Wu T.-K., Huang Y.-T. (2011). Mechanical grinding effect on thermodynamics and inclusion efficiency of loratadine-cyclodextrin inclusion complex formation. Carbohydr. Polym..

[32] Mennini N., Bragagni M., Maestrelli F., Mura P. (2014). Physico-chemical characterization in solution and in the solid state of clonazepam complexes with native and chemically-modified cyclodextrins. J. Pharm. Biomed. Anal..

[33] Cirri M., Maestrelli F., Mennini N., Mura P. (2009). Influence of the preparation method on the physical-chemical properties of ketoprofen-cyclodextrin-phosphatidylcholine ternary systems. J. Pharm. Biomed. Anal..

[34] Maestrelli F., Cecchi M., Cirri M., Capasso G., Mennini N., Mura P. (2009). Comparative study of oxaprozin complexation with natural and chemically-modified cyclodextrins in solution and in the solid state. J. Incl. Phenom. Macrocycl. Chem..

[35] Higashi K., Tozuka Y., Moribe K., Yamamoto K. (2010). Salicylic Acid/γ-Cydodextrin 2:1 and 4:1 Complex Formation by Sealed-Heating Method. J. Pharm. Sci..

[36] Cugovčan M., Jablan J., Lovrić J., Cinčić D., Galić N., Jug M. (2017). Biopharmaceutical characterization of praziquantel cocrystals and cyclodextrin complexes prepared by grinding. J. Pharm. Biomed. Anal..

[37] Brusnikina M., Silyukov O., Chislov M., Volkova T., Proshin A., Mazur A., Tolstoy P., Terekhova I. (2017). Effect of cyclodextrin complexation on solubility of novel anti-Alzheimer 1,2,4-thiadiazole derivative. J. Therm. Anal. Calorim..

[38] Prekodravac B., Damm M., Kappe C.O. (2011). Microwave-assisted forced degradation using high-throughput microtiter platforms. J. Pharm. Biomed. Anal..

[39] Colombo I., Grassi G., Grassi M. (2009). Drug mechanochemical actvation. J. Pharm. Sci..

[40] Jones W., Eddleston M.D. (2014). Introductory Lecture: Mechanochemistry, a versatile synthesis strategy for new materials. Faraday Discuss..

[41] Hernández J.G., Bolm C. (2017). Altering Product Selectivity by Mechanochemistry. J. Org. Chem..

[42] Friščić T. (2012). Supramolecular concepts and new techniques in mechanochemistry: Cocrystals, cages, rotaxanes, open metal–organic frameworks. Chem. Soc. Rev..

[43] Bikiaris D.N. (2011). Solid dispersions, Part I: Recent evolutions and future opportunities in manufacturing methods for dissolution rate enhancement of poorly water-soluble drugs. Expert Opin. Drug Deliv..

[44] Friščič T., Jones W. (2009). Recent advances in understanding the mechanism of cocrystal formation via grinding. Cryst. Growth Des..

[45] Aigner Z., Hassan H.B., Berkesi O., Kata M., Erős I. (2005). Thermoanalytical, FTIR and X-ray studies of gemfibrozil-cyclodextrin complexes. J. Therm. Anal. Calorim..

[46] Hasa D., Schneider Rauber G., Voinovich D., Jones W. (2015). Cocrystal Formation through Mechanochemistry: From Neat and Liquid-Assisted Grinding to Polymer-Assisted Grinding. Angew. Chem. Int. Ed..

[47] Cirri M., Bragagni M., Mennini N., Mura P. (2012). Development of a new delivery system consisting in “drug–In cyclodextrin–In nanostructured lipid carriers” for ketoprofen topical delivery. Eur. J. Pharm. Biopharm..

[48] Wongmekiat A., Tozuka Y., Oguchi T., Yamamoto K. (2002). Formation of fine drug particles by cogrinding with cyclodextrins. I. The use of β-cyclodextrin anhydrate and hydrate. Pharm. Res..

[49] Wongmekiat A., Tozuka Y., Oguchi T., Yamamoto K. (2003). Formation of fine drug particle by cogrinding with cyclodextrins: Part II. The influence of moisture condition during cogrinding process on fine particle formation. Int. J. Pharm..

[50] Wongmekiat A., Yoshimatsu S., Tozuka Y., Moribe K., Yamamoto K. (2006). Investigation of drug nanoparticle formation by co-grinding with cyclodextrins: Studies for indomethacin, furosemide and naproxen. J. Incl. Phenom. Macrocycl. Chem..

[51] Sun Y., Du L., Liu Y., Li X., Li M., Jin Y., Qian X. (2014). Transdermal delivery of the in situ hydrogels of curcumin and its inclusion complexes of hydroxypropyl-β-cyclodextrin for melanoma treatment. Int. J. Pharm..

[52] Zhou C., Li L., Liu Y., Wen S., Guo Y., Niu X. (2012). Study on the Inclusion Complex of Rutin/Sulfobutylether-β-Cyclodextrin. Adv. Mater. Res..

[53] Gu F.G., Wang Y., Meng G.D.L., Han H.B., Wu C.Z. (2012). Investigation of a fenofibrate-hydroxypropyl-β-cyclodextrin system prepared by a co-grinding method. Pharmazie.

[54] Li S., Zhai Y., Yan J., Wang L., Xu K., Li H. (2016). Effect of preparation processes and structural insight into the supermolecular system: Bisacodyl and β-cyclodextrin inclusion complex. Mater. Sci. Eng. C.

[55] Suzuki R., Inoue Y., Tsunoda Y., Murata I., Isshiki Y., Kondo S., Kanamoto I. (2015). Effect of γ-cyclodextrin derivative complexation on the physicochemical properties and antimicrobial activity of hinokitiol. J. Incl. Phenom. Macrocycl. Chem..

[56] Zhang J., He D., Tan Q., Wu M., Yang L., Ren Y., Liu J. (2013). Characterization, activity, and computer modeling of a molecular inclusion complex containing rifaldazine. Int. J. Nanomed..

[57] Aigner Z., Berkesi O., Farkas G., Szabó-Révész P. (2012). DSC, X-ray and FTIR studies of a gemfibrozil/dimethyl-β-cyclodextrin inclusion complex produced by co-grinding. J. Pharm. Biomed. Anal..

[58] He D., Deng P., Yang L., Tan Q., Liu J., Yang M., Zhang J. (2013). Molecular encapsulation of rifampicin as an inclusion complex of hydroxypropyl-β-cyclodextrin: Design; characterization and in vitro dissolution. Colloids Surf. B Biointerfaces.

[59] Ogawa N., Higashi K., Nagase H., Endo T., Moribe K., Loftsson T., Yamamoto K., Ueda H. (2010). Effects of cogrinding with β-cyclodextrin on the solid state fentanyl. J. Pharm. Sci..

[60] Anzai K., Mizoguchi J., Yanagi T., Hirayama F., Arima H., Uekama K. (2007). Improvement of dissolution properties of a new Helicobacter pylori eradicating agent (TG44) by inclusion complexation with beta-cyclodextrin. Chem. Pharm. Bull..

[61] Mura P., Furlanetto S., Cirri M., Maestrelli F., Corti G., Pinzauti S. (2005). Interaction of naproxen with ionic cyclodextrins in aqueous solution and in the solid state. J. Pharm. Biomed. Anal..

[62] Mura P., Bettinetti G.P., Cirri M., Maestrelli F., Sorrenti M., Catenacci L. (2005). Solid-state characterization and dissolution properties of Naproxen-Arginine-Hydroxypropyl-β-cyclodextrin ternary system. Eur. J. Pharm. Biopharm..

[63] Caira M.R., Dodds D.R., Nassimbeni L.R. (2002). Polymorphism and Cyclodextrin Inclusion of Salbutamol Laurate. J. Therm. Anal. Calorim..

[64] Bettinetti G., Mura P., Faucci M.T., Sorrenti M., Setti M. (2002). Interaction of naproxen with noncrystalline acetyl beta- and acetyl gamma-cyclodextrins in the solid and liquid state. Eur. J. Pharm. Sci..

[65] Mitrevej A., Sinchaipanid N., Junyaprasert V., Warintornuwat L. (1996). Effect of grinding of beta-cyclodextrin and glibenclamide on tablet properties. 1. In vitro. Drug Dev. Ind. Pharm..

[66] Fumić B., Jablan J., Cinčić D., Zovko Končić M., Jug M. (2017). Cyclodextrin encapsulation of daidzein and genistein by grinding: Implication on the glycosaminoglycan accumulation in mucopolysaccharidosis type II and III fibroblasts. J. Microencapsul..

[67] Majewska K., Skwierawska A., Kamińska B., Prześniak-Welenc M. (2018). Improvement of opipramol base solubility by complexation with β-cyclodextrin. Supramol. Chem..

[68] Ali H.R.H., Saleem I.Y., Tawfeek H.M. (2016). Insight into inclusion complexation of indomethacin nicotinamide cocrystals. J. Incl. Phenom. Macrocycl. Chem..

[69] Borba P.A.A., Pinotti M., Andrade G.R.S., Da Costa N.B., Olchanheski L.R., Fernandes D., De Campos C.E.M., Stulzer H.K. (2015). The effect of mechanical grinding on the formation, crystalline changes and dissolution behaviour of the inclusion complex of telmisartan and β-cyclodextrins. Carbohydr. Polym..

[70] Ramos A.I., Braga T.M., Silva P., Fernandes J.A., Ribeiro-Claro P., Lopes M.D.F.S., Paz F.A.A., Braga S.S. (2013). Chloramphenicol·cyclodextrin inclusion compounds: Co-dissolution and mechanochemical preparations and antibacterial action. CrystEngComm.

[71] Zong W., Bi S. The preparation and characterization of inclusion complex of ursolic acid with γ-cyclodextrin. Proceedings of the 2011 7th International Conference on MEMS NANO, and Smart Systems (ICMENS 2011).

[72] Corti G., Capasso G., Maestrelli F., Cirri M., Mura P. (2007). Physical-chemical characterization of binary systems of metformin hydrochloride with triacetyl-β-cyclodextrin. J. Pharm. Biomed. Anal..

[73] Cirri M., Maestrelli F., Furlanetto S., Mura P. (2004). Solid-state characterization of glyburide-cyclodextrin co-ground products. J. Therm. Anal..

[74] Mura P., Faucci M.T., Maestrelli F., Furlanetto S., Pinzauti S. (2002). Characterization of physicochemical properties of naproxen systems with amorphous β-cyclodextrin-epichlorohydrin polymers. J. Pharm. Biomed. Anal..

[75] Mura P., Faucci M.T., Manderioli A., Bramanti G. (2001). Multicomponent systems of econazole with hydroxyacids and cyclodextrins. J. Incl. Phenom..

[76] Mura P., Faucci M.T., Parrini P.L., Furlanetto S., Pinzauti S. (1999). Influence of the preparation method on the physicochemical properties of ketoprofen-cyclodextrin binary systems. Int. J. Pharm..

[77] Jablan J., Bačić I., Kujundžić N., Jug M. (2013). Zaleplon co-ground complexes with natural and polymeric β-cyclodextrin. J. Incl. Phenom. Macrocycl. Chem..

[78] Mura P., Faucci M.T., Bettinetti G.P. (2001). The influence of polyvinylpyrrolidone on naproxen complexation with hydroxypropyl-β-cyclodextrin. Eur. J. Pharm. Sci..

[79] Jug M., Kosalec I., Maestrelli F., Mura P. (2011). Analysis of triclosan inclusion complexes with β-cyclodextrin and its water-soluble polymeric derivative. J. Pharm. Biomed. Anal..

[80] Jug M., Mennini N., Kövér K.E., Mura P. (2014). Comparative analysis of binary and ternary cyclodextrin complexes with econazole nitrate in solution and in solid state. J. Pharm. Biomed. Anal..

[81] Jug M., Maestrelli F., Bragagni M., Mura P. (2010). Preparation and solid-state characterization of bupivacaine hydrochloride cyclodextrin complexes aimed for buccal delivery. J. Pharm. Biomed. Anal..

[82] Shah B., Kakumanu V.K., Bansal A.K. (2006). Analytical techniques for quantification of amorphous/crystalline phases in pharmaceutical solids. J. Pharm. Sci..

[83] Vogt F.G., Strohmeier M. (2012). 2D Solid-State NMR Analysis of Inclusion in Drug–Cyclodextrin Complexes. Mol. Pharm..

[84] Redenti E., Peveri T., Zanol M., Ventura P., Gnappi G., Montenero A. (1996). A study on the differentiation between amorphous piroxicam:β-cyclodextrin complex and a mixture of the two amorphous components. Int. J. Pharm..

[85] Anzai K., Kono H., Mizoguchi J., Yanagi T., Hirayama F., Arima H., Uekama K. (2006). Two-dimensional 13C–1H heteronuclear correlation NMR spectroscopic studies for the inclusion complex of cyclomaltoheptaose (β-cyclodextrin) with a new Helicobacter pylori eradicating agent (TG44) in the amorphous state. Carbohydr. Res..

[86] Takacs L. (2013). The historical development of mechanochemistry. Chem. Soc. Rev..

[87] Bettinetti G., Sorrenti M., Negri A., Setti M., Mura P., Melani F. (1999). Interaction of naproxen with alpha-cyclodextrin and its noncyclic analog maltohexaose. Pharm. Res..

[88] Veiga M.D., Ahsan F., Merino M. (1999). Differential scanning calorimetry as an analytical tool in determining the interaction between drug and cyclodextrin. J. Therm. Anal. Calorim..

[89] Mixer Mill MM 200–RETSCH–High Performance and Great Flexibility. https://www.retsch.com/products/milling/ball-mills/mixer-mill-mm-200/function-features/.

[90] 8000D Mixer/Mill—Dual Clamp, High-Energy Ball Mill, Mechanical Alloying. Grinds Hard Samples|SPEX SamplePrep. https://www.spexsampleprep.com/8000D-mixermill.

[91] Howard J.L., Cao Q., Browne D.L. (2018). Mechanochemistry as an emerging tool for molecular synthesis: What can it offer?. Chem. Sci..

[92] Mura P., Faucci M.T., Parrini P.L. (2001). Effects of grinding with microcrystalline cellulose and cyclodextrins on the ketoprofen physicochemical properties. Drug Dev. Ind. Pharm..

[93] Lin S.Y., Hsu C.H., Sheu M.T. (2010). Curve-fitting FTIR studies of loratadine/hydroxypropyl-β-cyclodextrin inclusion complex induced by co-grinding process. J. Pharm. Biomed. Anal..

[94] Corvi Mora P., Cirri M., Allolio B., Carli F., Mura P. (2003). Enhancement of Dehydroepiandrosterone Solubility and Bioavailability by Ternary Complexation with α-Cyclodextrin and Glycine. J. Pharm. Sci..

[95] Mennini N., Maestrelli F., Cirri M., Mura P. (2016). Analysis of physicochemical properties of ternary systems of oxaprozin with randomly methylated-ß-cyclodextrin and L-arginine aimed to improve the drug solubility. J. Pharm. Biomed. Anal..

[96] James S.L., Adams C.J., Bolm C., Braga D., Collier P., Frisčc T., Grepioni F., Harris K.D.M., Hyett G., Jones W. (2012). Mechanochemistry: Opportunities for new and cleaner synthesis. Chem. Soc. Rev..

[97] Planetary Ball Mill PM 200–RETSCH–Short Grinding Times. https://www.retsch.com/products/milling/ball-mills/planetary-ball-mill-pm-200/function-features/.

[98] Iwata M., Fukami T., Kawashima D., Sakai M., Furuishi T., Suzuki T., Tomono K., Ueda H. (2009). Effectiveness of mechanochemical treatment with cyclodextrins on increasing solubility of glimepiride. Pharmazie.

[99] Zheng M., Tang W., Kong R., Zhu X. (2017). Inclusion Complex of α -Lipoic Acid Containing Alkalizer for Improving the Solubility and Stability Prepared by Co-grinding. Indian J. Pharm. Sci..

[100] Li S., Lin X., Xu K., He J., Yang H., Li H. (2017). Co-grinding Effect on Crystalline Zaltoprofen with β-cyclodextrin/Cucurbit[7]uril in Tablet Formulation. Sci. Rep..

